# Mild psoriasis as a suitable model for proof‐of‐mechanism in a phase 1B setting: Results from a double‐blind placebo‐controlled trial with guselkumab

**DOI:** 10.1002/bcp.70179

**Published:** 2025-07-28

**Authors:** Jannik Rousel, Menthe E. Bergmans, Lisa J. Bruijnincx, Sissi Lin, Tessa Niemeyer‐van der Kolk, Roman Bohoslavsky, Yalçin Yavuz, Naomi B. Klarenbeek, Joke A. Bouwstra, Robert Rissmann, Martijn B. A. van Doorn, the Next‐Generation ImmunoDermatology Consortium (NGID)

**Affiliations:** ^1^ Centre for Human Drug Research Leiden the Netherlands; ^2^ Leiden Academic Centre for Drug Research Leiden University Leiden the Netherlands; ^3^ Leiden University Medical Centre Leiden the Netherlands; ^4^ Erasmus Medical Centre Rotterdam the Netherlands

**Keywords:** imaging, phase I, psoriasis, randomized controlled trial

## Abstract

**Aims:**

Early clinical development of novel psoriasis therapies is hampered by a decreasing number of moderate‐to‐severe psoriasis patients eligible for participation in trials since efficacious treatments, such as biologics, have become widely available. The aim of this study was to establish mild psoriasis patients as a suitable alternative trial population since these patients are generally not eligible for these treatments.

**Methods:**

A randomized double‐blind controlled trial was performed in 20 mild psoriasis patients (psoriasis area and severity index ≤5), randomized 3:1 to guselkumab 100 mg or placebo, and 5 moderate‐to‐severe psoriasis patients (psoriasis area and severity index ≥10) on guselkumab 100 mg. Clinical scoring was performed over 24 weeks and substantiated with multimodal imaging comprising multispectral imaging, optical coherence tomography and laser speckle contrast imaging.

**Results:**

Clinician‐reported outcomes demonstrated significant treatment effects compared to placebo in mild patients. Focusing on a target plaque, severity scores significantly decreased during treatment with guselkumab only. Imaging demonstrated significant decreases in erythema, maximal height within‐lesion and cutaneous perfusion compared to placebo. Plaques of mild and moderate‐to‐severe patients did not differ at baseline and showed similar treatment responses.

**Conclusion:**

Clinical scoring and multimodal lesion monitoring enabled the detection of a clear treatment effect of guselkumab in mild psoriasis patients. Although this trial was not powered to demonstrate equivalence between the severity groups, our results indicate that the treatment responses follow the same trend in mild and moderate‐to‐severe patients with a high degree of similarity. Therefore, mild patients can be considered a suitable study population for early phase proof‐of‐concept trials.

**What is already known about this subject**

- Research into new therapies for psoriasis is mainly conducted in moderate‐to‐severe patients.
- The availability of efficacious treatments reduces their willingness to partake in clinical trials, which hampers drug development.
- Patients with mild disease are often ineligible for these treatments and could therefore present suitable alternatives presuming similar treatment responses are demonstrated.

**What does this study add**

- Supported by multimodal imaging, this study shows treatment responses can be objectively established in mild patients with a high degree of similarity to moderate‐to‐severe patients.
- This underlines that mild patients present a suitable alternative for moderate‐to‐severe patients in clinical trials.
- This provides a more ethical and robust framework for future trials.

## 1 INTRODUCTION

Plaque psoriasis is a common chronic skin disease that affects 2–3% of the world's population.^1^, ^2^ The quality of life of moderate‐to‐severe psoriasis patients has increased tremendously in recent years due to the emergence of biologic therapies that are safer and more efficacious compared to conventional systemic therapies such as methotrexate and cyclosporine.^3^ As a result, the number of moderate‐to‐severe psoriasis patients in western countries who are eligible for clinical trials and are willing to abstain from therapy awaiting enrolment has markedly decreased. Besides, the requirement for month(s) long washout periods to demonstrate clinical efficacy for patients on systematic therapies and the randomization risk to placebo can result in additional burden.^4^, ^5^ This impacts the development of innovative and potentially curative treatments as participation of psoriasis patients is still needed in their clinical development.

Being the intended treatment group for biologics, clinical trials have been primarily conducted in this shrinking moderate‐to‐severe psoriasis population. However, up to 80% of psoriasis patients is estimated to have a milder form of psoriasis with limited skin involvement.^6^, ^7^ In this population, treatment with conventional systemic treatments or biologics is deemed unwarranted due to unfavourable risk–benefit and cost–benefit ratios.^8^, ^9^ Instead, their treatment options remain limited to the use of topical corticosteroids that are associated with poor efficacy and rapid relapse upon cessation of treatment.^10^, ^11^ This group of patients with negligible systemic exposure, active symptoms and limited burden could be valuable candidates for clinical trials in psoriasis. Additionally, psoriasis occurs regularly alongside other immune mediated diseases that share some, if not the majority, of the pathogenic drivers of psoriasis.^12^ Mild psoriasis patients might therefore also serve as a model population for proof‐of‐concept first‐in‐patient trials extending beyond plaque psoriasis.

However, it is critical that results obtained in this mild population translate well to moderate‐to‐severe patients and reflect trial outcomes similarly. While differences in the transcriptomic profile of plaques between these groups have been observed, they appear rather limited and do not result in major differences in treatment responses.^13^, ^14^ Rather, the challenge may lie in reliable detection of treatment responses as the widely adopted psoriasis area and severity index (PASI) has low sensitivity when disease severity is low.^15^, ^16^, ^17^ This might be resolved by adapting an objective and data‐rich multimodal approach that extends past physician reported outcomes.^18^ Imaging has been extensively applied in dermatology to monitor treatment responses and its digitalized nature precludes inter‐ and intraobserver variability associated with physician‐reported outcomes, which might yield higher sensitivity regardless of severity.^19^, ^20^ However, their applicability in psoriasis should first be addressed before these methods can be used.

The objective of this study was to demonstrate that the clinical response of mild patients can reliably be detected in a trial setting. We have conducted a placebo‐controlled double‐blind clinical trial with the anti‐interleukin‐23 monoclonal antibody guselkumab, in which we characterized the treatment‐response of mild patients with at least one moderate target plaque and include several moderate‐to‐severe patients as reference. Classic PASI‐based endpoints were complemented with a multimodal imaging toolbox to bolster confidence for the small group sizes employed in Phase I/II trials. This trial can establish mild psoriasis patients as relevant model for moderate‐to‐severe patients with methodology that can be readily adopted in upcoming early‐stage clinical trials. Moreover, highlighting similar responses in mild psoriasis patients further highlights the applicability of biologic therapy in these patients.

## 2 METHODS

A more extensive method section is included in the supplementary material. Key protein targets and ligands in this article are hyperlinked to corresponding entries in http://www.guidetopharmacology.org, and are permanently archived in the Concise Guide to PHARMACOLOGY 2023/24.^21^

### 2.1 Study design

This exploratory, single‐centre, double‐blinded and placebo‐controlled randomized clinical trial registered under ClinicalTrials.gov Identifier NCT03688971 was performed from September 2020 to January 2023 conform the Declaration of Helsinki principles at the Centre for Human Drug Research, in Leiden, the Netherlands. Ethical approval was obtained from the institutional review board METC Brabant (Tilburg, the Netherlands). Written informed consent was obtained from participants before any study‐related procedures were performed. General health was evaluated at screening and psoriasis‐specific aspects were evaluated for patients. Patients were only included when presenting at screening with ≥1 lesion of lesion severity score (LSS) ≥ 6 and with a PASI score of ≤5 (mild psoriasis group) or ≥10 (moderate‐to‐severe psoriasis group). All in‐ and exclusion requirements, including washouts, for healthy controls and patients are included in the supplemental material. Patients were randomized 3:1 to standard‐of‐care induction therapy with guselkumab 100 mg (GUS) subcutaneously or placebo (PLA). No concomitant medication was allowed. Patients visited the Centre for Human Drug Research for visits 2, 4, 8, 12, 16 and 24 weeks after first dose, with 2 more 100 mg doses given at week 4 and 12. Clinical scoring and imaging were performed on the same lesion with LSS ≥ 6 at baseline at every visit.

### 2.2 Clinical scoring

Three staff members performed all clinical scoring after training by, and subsequent authorization of, a licensed dermatologist. Psoriasis severity was evaluated using PASI and Physicians Global Assessment (PGA) scoring as commonly applied.^22^, ^23^ The percentage body surface area affected (%BSA) was estimated per body region by referencing the patient's palm as 1% BSA. This enabled the use of the PASI‐high discrimination (PASI‐HD), which subdivides the lowest categorical area value for the PASI (<10%BSA = 1) in tenths (e.g., 8.5%BSA = 0.8).^15^ Single lesions were scored using a 5‐point grading system for erythema, induration and scaling for psoriasis (0–4; none–very severe), with the sum representing the LSS.^24^

### 2.3 Total body photography

Automated total body photography was performed using the ATBM system (FotoFinder Systems GmbH, Bad BirnBach, Germany) with subjects photographed from all sides. Subjects were photographed from the sides, back and front while dressed in disposable underwear covering solely the genitalia. Subjects adopted standard poses as prompted by the ATBM system. Flash photography was performed in front of the same dark blue backdrop under standardized lighting conditions. The proprietary PASIvision Universe software 2.0.41.20 was used to obtain digital PASI (dPASI) scores. It automatically combines the 4 pictures for each side and overlays them with a filter indicating affected skin area and calculates the erythema, scaling, induration and body surface area.^25^ Besides a minimal correction for affected surface area by the operator, no other changes were made before submitting the scores yielding a computerized dPASI as previously applied by Fink *et al*.^26^

### 2.4 Optical coherence tomography

The Vivosight Dx Optical Coherence Tomography (OCT) software (Michelson Diagnostics, Kent, UK) was used to obtain a scan over a 6 × 6 mm area of skin. Skin roughness was extracted from measurement files after analysis through the proprietary Vivosight Tools analysis software (Michelson Diagnostics). Epidermal thickness was determined by analysing the exported scans containing 120 frames using ImageJ and averaging 36 depth measurements at the centre and both sides every 10 frames.

### 2.5 Multispectral imaging

An Antera 3D (Miravex, Dublin, Ireland) was used to capture multispectral 3D images of the skin. Relevant parameters were extracted from the software after analysing a 40‐mm diameter circle of skin using the Antera 3D proprietary software.

### 2.6 Colorimetry

The degree of redness is determined using a Cortex DSM‐3 (Aalborg, Denmark). Redness is determined by averaging the CIElab A*‐value of 3 different overlapping measurements. The A*‐value represents the degree of redness from a scale of 0–60.1.

### 2.7 Laser speckle contrast imaging

The cutaneous microcirculation is captured using the Pericam PSI imager (Perimed, Järfälla, Sweden). The device is calibrated before the first measurement of the day. Perfusion is determined within a 40‐mm diameter circular region of interest over a continuous recording of 30 s. Prior to measurement, subjects acclimatized a minimum of 15 min to the temperate and humidity‐controlled room (humidity <60%, temperature 22 ± 2°C).

### 2.8 Statistics

Group sizes were determined based on PASI‐90 responses from Phase III guselkumab trials.^27^, ^28^ Heat map and baseline comparisons using an unpaired 1‐way ANOVA with multiple comparisons with Tukey's *posthoc* tests were made in Prism 9.0 (GraphPad, Software, Boston, MA, USA). Longitudinal effects were analysed with a mixed model of repeated measures (ANCOVA), with group (severity‐treatment: mild‐GUS, moderate‐to‐severe‐GUS, mild‐PLA), time, and group‐by‐time as fixed factors and patients as random factor, assuming a variance component variance–covariance matrix in SAS version 9.4 (SAS Institute, Cary, NC, USA). Contrasts were reported in respect to changes from baseline for each group, and between the mild‐GUS and mild‐PLA groups. Common within‐individual associations for paired measures were determined using Rmcorr in R Statistical Software (version 4.1.2, R Core Team 2021).^29^ For conciseness, associated means, confidence‐intervals and *P*‐values are listed in Table S3. In‐text values represent mean ± standard deviations. *P*‐values are denoted by *: *P* ≤ .05, **: *P* ≤ .01, ***: *P* ≤ .001.

## 3 RESULTS

Of the 78 patients screened for eligibility, 21 patients with mild and 6 patients with moderate‐to‐severe psoriasis were included into the study (Figure S1). Participating patients were predominantly white with a Fitzpatrick skin type of II and III (Table S1). One patient dropped out, citing incompatibility with the protocol before randomization; 15/20 mild and 5/6 moderate‐to‐severe patients were allocated to guselkumab treatment. The single moderate‐to‐severe patient receiving placebo is only included in baseline comparisons together with 10 healthy controls. All dosed subjects completed the study without any serious adverse events (Table S2). Efficacy and tolerability in the mild study population was reported previously.^30^

### 3.1 Efficacy in mild and moderate‐to‐severe patients as assessed by clinical scoring

**FIGURE 1:**
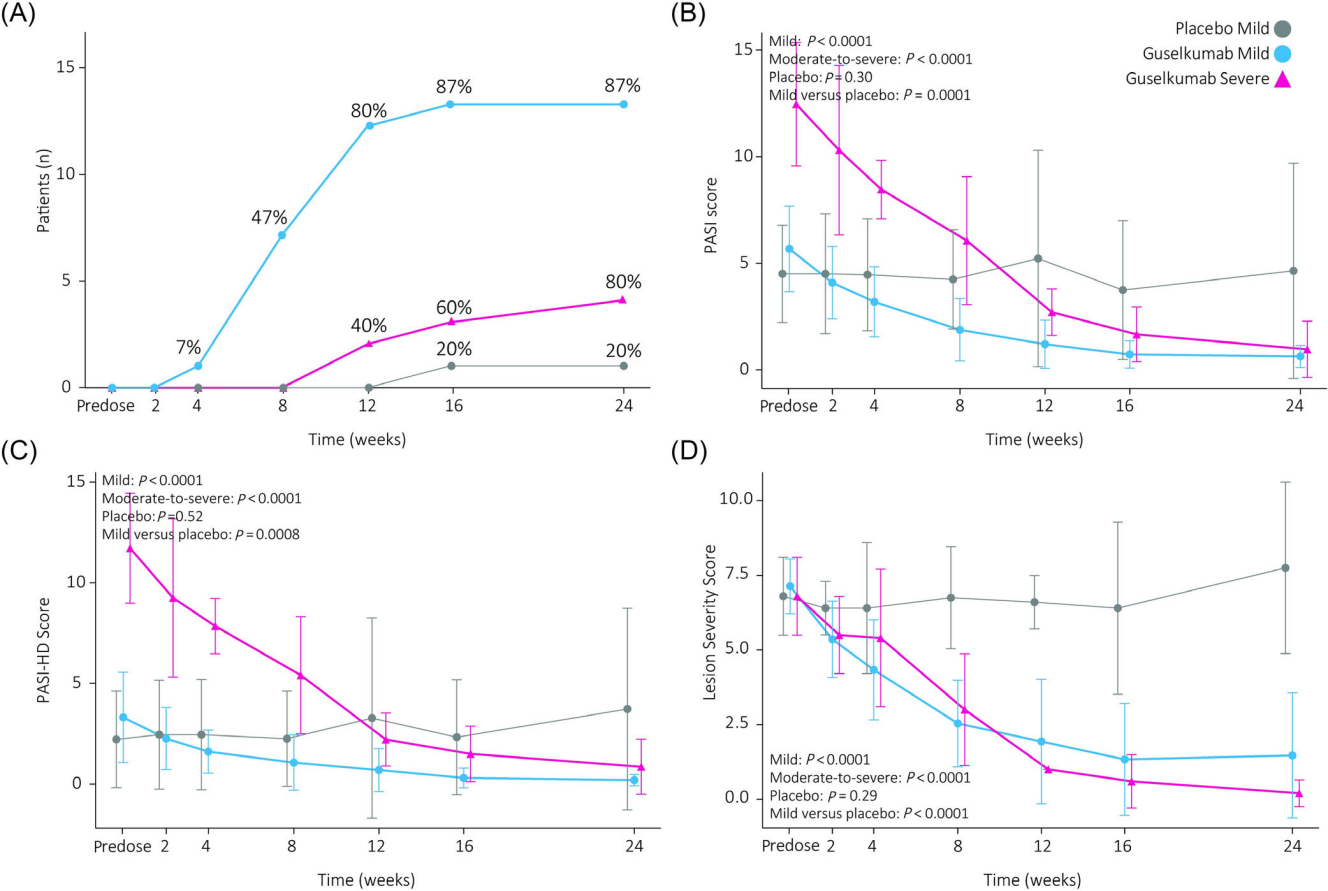
Clinical scoring of psoriasis severity. The number of subjects reaching a physicians global assessment rating of clear or almost clear (0/1) with the number of patients on the Y‐axis and percentages in the graph indicating the fraction of patients reaching physicians global assessment 0/1 (A). The psoriasis area and severity index (PASI; B) and the PASI–high discrimination (PASI‐HD), which has a higher sensitivity below 10% involved body surface area (C) shows significant decreases in psoriasis severity in the guselkumab treated groups. This is reiterated by monitoring the lesion severity score of a representative but moderate target plaque of at least lesion severity score ≥ 6 at baseline over time (D). Graphs show the mean and standard deviations.

Clinician‐reported endpoints show evident treatment effects (Figure 1). Severity scoring with the simple 5‐point PGA‐scale showed 13/15 mild patients and 4/5 moderate‐to‐severe patients randomized to guselkumab obtained PGA 0/1 (clear/almost clear) compared to 1/5 mild patients in the placebo group with all patients having PGA ≥ 2 at baseline.

**FIGURE 2:**
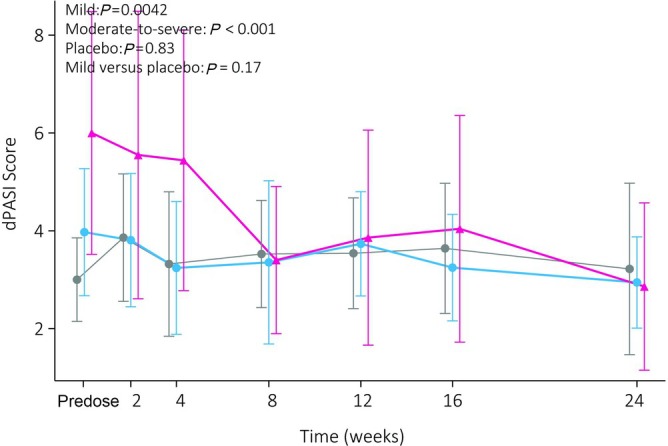
The digital psoriasis area and severity index (dPASI) over time. The mean and standard deviation is shown. Although clinician reported outcomes demonstrate significant differences compared to placebo, the dPASI is unable to replicate this.

PASI scores were higher in the moderate‐to‐severe group compared to the mild group (12.82 ± 2.7 *vs*. 5.4 ± 2.1, *P <* .0001). PASI scores decreased significantly compared to baseline for both guselkumab groups, but not in the placebo group. Mild patients treated with guselkumab showed a significant decrease compared to those on placebo. PASI‐75, PASI‐90 and PASI‐100 responses in the guselkumab groups were obtained by 15/15 (100%), 8/15 (53%) and 6/15 (40%) in the mild group and by 5/5 (80%), 4/5 (60%) and 1/5 (20%) in the moderate‐to‐severe group. Of note, the dPASI was able to detect a significant decrease from baseline in the treatment groups, but was unable to establish effects compared to placebo and correlated poorly with the physician‐performed PASI (*r*_rm_: 0.45, Figures 2 and S2).

Tailoring the PASI score to the mild population by enhancing sensitivity at lower %BSA, the PASI‐HD and PASI scores at baseline showed little difference in the moderate‐to‐severe group (PASI: 12.5 ± 0.9, PASI‐HD: 11.7 ± 2.7) but were affected in the mild patient group (PASI: 5.6 ± 2.0, PASI‐HD: 2.2 ± 2.4). In both groups, a significant treatment effect was observed upon guselkumab treatment. As with regular PASI scoring, a significant decrease compared to placebo was observed for mild patients. PASI‐HD treatment responses exceeded those determined by PASI, with 15/15 (100%), 12/15 (80%) and 6/15 (40%) of mild guselkumab‐treated patients and 5/5 (100%), 4/5 (80%) and 1/5 (20%) of moderate‐to‐severe guselkumab treated patients obtaining PASI‐HD‐75, PASI‐HD‐90 and PASI‐HD‐100 scores, respectively.

When focussing on the resolution of a single lesion, the cumulative LSS was similar at baseline between both severity groups (mild: 7.1 ± 1.0, moderate‐to‐severe: 6.7 ± 1.2, *P =* .97) in contrast to PASI. After treatment, guselkumab significantly reduced LSS compared to baseline. For the mild patients, this decrease was significant compared to placebo.

### 3.2 Objective confirmation of physician‐based treatment responses in patients

Analysis of skin roughness (R_a_) by multispectral imaging and OCT showed a significantly higher roughness at lesional skin compared to nonlesional skin in mild patients, but no other significant baseline differences in skin roughness were observed compared to controls or within the moderate‐to‐severe group. Longitudinal monitoring showed roughness was significantly reduced after guselkumab treatment compared to baseline in both guselkumab groups for OCT and in the mild guselkumab group for multispectral imaging. However, no significant differences compared to placebo were observed for both analysis (Figure S3).

**FIGURE 3:**
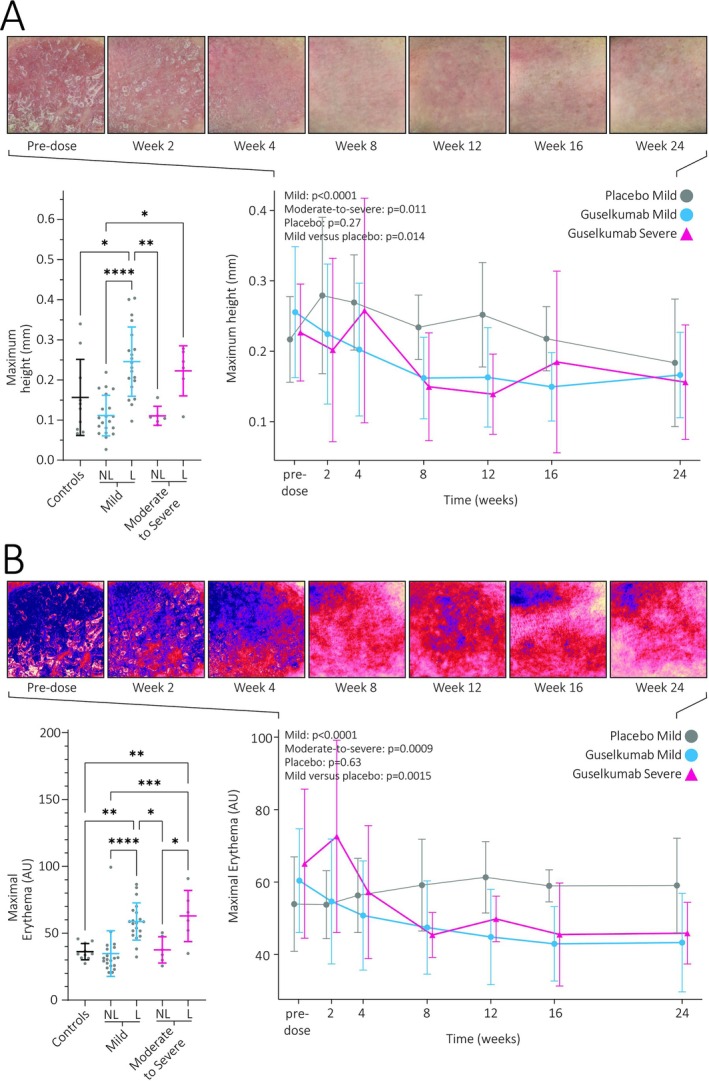
Multispectral imaging for the determination of superficial roughness (A) and erythema (B). Comparisons between healthy control skin, nonlesional skin and lesional skin are made at baseline, with the lesional skin of patients monitored further throughout the trial. The time course has been supplemented with visual output of the modalities, showing the effect of guselkumab in the same subject. Texture and erythema images of 3 additional subjects are presented in the supplemental material in Figures S5 and S6. Graphs show the mean and standard deviations. AU, arbitrary units; L, lesional; NL, nonlesional. [Correction added on 4 August 2025, after first online publication: Figure 3 has been updated in this version.]

Maximum height (R_t_) within the analysed region proved significantly higher in lesional skin compared to nonlesional skin and that of controls for mild patients (Figures 3, S5 and S6). In moderate‐to‐severe patients, R_t_ in lesional skin was neither increased compared to their nonlesional skin or that of controls. However, lesional skin showed evident decreases in R_t_ over time for both guselkumab‐treated groups. After treatment, R_t_ was significantly decreased compared to placebo in mild patients.

Comprising a pertinent part of clinical scoring, digitalization of erythema grading might constitute an applicable outcome measure. The A*‐CIELAB value from single‐point colorimetry indicated significantly more erythema at lesional compared to nonlesional skin. However, no significant differences between controls and lesional skin were observed. Additionally, monitoring lesional A* did not result in significant reductions compared to baseline or placebo (Figure S4).

By contrast, multispectral imaging was able to highlight increased maximum erythema over a bigger region of interest in lesional skin compared to controls and compared to nonlesional skin (Figure 3). Lesional skin did not significantly differ between the 2 treatment groups at baseline. Maximal erythema decreased during guselkumab treatment, resulting in significantly lower erythema compared to baseline regardless of severity. This resulted in a significantly decreased erythema compared to placebo for mild patients. Similar results were obtained when monitoring the delta A*‐value but not with average A* (Figure S4).

### 3.3 Subsurface changes in skin perfusion and epidermal thickness support visually observed treatment effects

**FIGURE 4:**
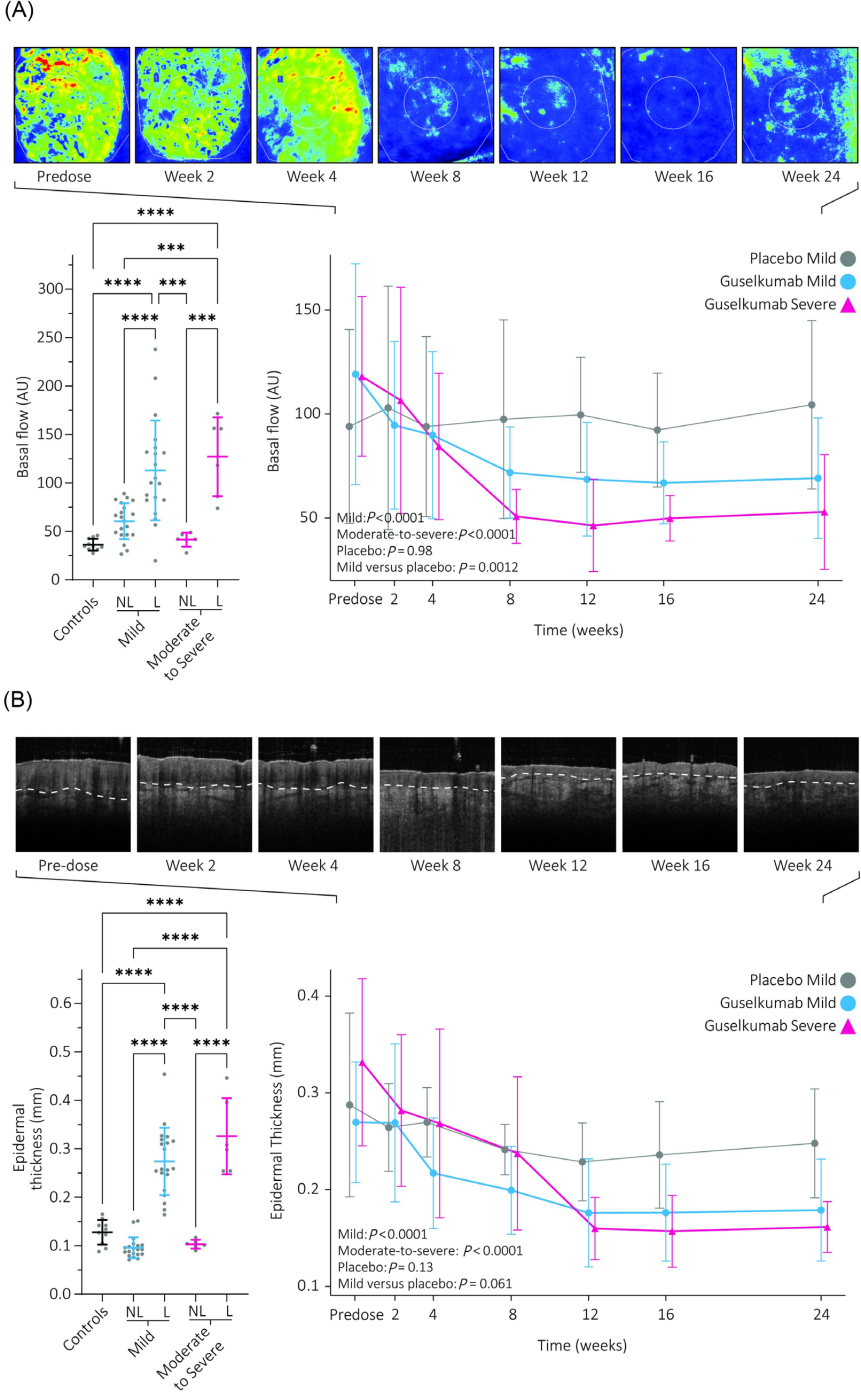
Baseline differences and longitudinal monitoring of the cutaneous perfusion in target lesions by laser speckle contrast imaging (A) and epidermal thickness by optical coherence tomography (B). The effect over time is supplemented with the visual output of the device with the degree in cutaneous perfusion in a gradient from blue to green to red, with red indicating the highest degree of perfusion. The dermal–epidermal junction is indicated with a dotted line in the optical coherence tomography images. Images shown pertain to the same lesion from the same patient on guselkumab as shown earlier, lesions of additional patients are included in the supplementary methods in Figures S7 and S8. Graphs show the mean and standard deviations. AU, arbitrary units; L, lesional; NL, nonlesional.

Cutaneous perfusion is enhanced in lesional skin compared to nonlesional and control skin (Figures 4, S7). Lesional perfusion is not significantly higher in skin of moderate‐to‐severe patients compared to mild patients. During guselkumab treatment, basal flow declined significantly compared to baseline in both mild and moderate‐to‐severe patients but not in the placebo group. Compared directly to placebo in the mild group, this remained a significant decrease.

Optical coherence tomography showed epidermal thickness was increased in both mild and moderate‐to‐severe patients when compared to their nonlesional skin and controls at baseline (Figures 4, S8). Over time, epidermal thickness decreased significantly in both actively treated groups but not in the placebo group. However, no significant difference was observed when comparing mild patients on guselkumab to placebo.

### 3.4 Clinical and digital endpoints correlate

Summarizing, evident treatment effects are observed throughout the mild and moderate‐to‐severe population (Table 1). As outlined in Figure 1, clinical scoring by PASI, PASI‐HD and LSS provides strong indications of disease remission in both treatment groups with decreases of >80%. With the exception of the R_t_, which decreases below the average of healthy controls in moderate‐to‐severe patients, imaging endpoints show a smaller reduction compared to clinical scores and are indicative of a placebo effect as decreases are observed for epidermal thickness and R_t_ in the placebo group. The repeated measures correlation was high among the different clinical endpoints (r_rm_ > 0.7) but moderate between clinical scores and objective endpoints (0.37 < r_rm_ < 0.71; Tables S4, S5). Coefficients increased marginally only for maximal height when using the applicable LSS subscores instead of total LSS (induration–epidermal thickness, erythema–maximal erythema and scaling–maximal height).

**TABLE 1:** Degree of the normalization in clinical and imaging parameters after 16 weeks of guselkumab treatment. The percentage decrease or increase from baseline is indicated for each group with −100% indicating full normalization to a healthy state for clinical parameters (PASI, PASI‐HD, lesion severity score, dPASI) or to the level observed in healthy control skin for imaging parameters (cutaneous perfusion, epidermal thickness, maximal erythema and maximal height. The degree a parameter normalizes, remains similar or worsens compared to controls is indicated by a green‐white‐red gradient, respectively. PASI: psoriasis area and severity index, PASI‐HD: PASI–high discrimination, dPASI: digital PASI.

	Placebo	Mild	Moderate to severe
PASI score	−4.26	−87.72	−93.46
PASI‐HD score	+31.46	−94.87	−94.03
Lesion severity score	+8.33	−81.14	−97.5
dPASI sore	+94.11	−0.79	−29.61
Cutaneous perfusion	+18.03	−60.33	−79.62
Epidermal thickness	−24.8	−63.97	−83.47
Maximal erythema	+29.11	−70.84	−66.56
Maximal height	−55.05	−90.04	−100.56

## 4 DISCUSSION

By complementing traditional clinical endpoints with objective digitalized endpoints, we have shown how treatment responses can be readily observed in a cohort of 20 mild psoriasis patients and can differentiate between active treatment and placebo. Although this trial was not powered to compare the treatment response in mild patients directly with their moderate‐to‐severe counterparts, characterizing these populations at baseline and subsequently following guselkumab‐induced treatment responses adequately indicated differences were small.

As a direct result from the in‐ and exclusion criteria, significant baseline differences in PASI‐assessed disease severity were apparent between the mild and moderate‐to‐severe patients, in line with the European Medicines Agency classification of these patient groups.^31^ However, it has to be noted that the definition of mild patients is subject to debate and is influenced by more factors than PASI alone.^32^ It has been described that insensitivity of the PASI at low disease severity might complicate the effective use of PASI‐scoring in mild patients as low baseline scores require near‐complete clearance to achieve PASI‐75 and PASI‐90 responses.^15^, ^16^ However, significant PASI‐responses were observed for mild patients, indicating that this scoring system remains sufficiently powered to detect differences in populations with low baseline scores. Although PASI‐75 and PASI‐90 responses in our mild population were comparable to earlier phase III studies in moderate‐to‐severe patients,^27^, ^28^ implementation of the PASI‐HD enhanced responses that might more appropriately reflect disease remission in this group as PASI has been shown to underrepresent actual clinical effects.^33^ All patients in this study were recruited based on the presence of at least 1 moderate psoriasis plaque to allow for an evident plaque to monitor during treatment. In practice, this resulted in a similar LSS at baseline for both severity groups with different total PASI scores, which illustrates how the extent of psoriasis impacts the latter.^33^ In terms of single lesions, additional measures did not show any significant differences between mild and moderate‐to‐severe patients and nonlesional skin appeared not significantly different from that of controls.

The cutaneous morphology of lesional skin normalized during treatment. Expected increases in lesional roughness, caused by the presence of scaling, were hard to establish and might have resulted from insufficient sensitivity to detect fine scaling.^34^ Instead, maximum height proved to be a suitable endpoint that differentiated the patient and control groups at baseline and active treatment from placebo. Evidently tied to the psoriatic phenotype, redness decreased over time when expressed as maximal erythema and delta A*. Objectively quantifying erythema has been exploited to monitor disease remission before in psoriasis^35^ and other indications,^20^, ^36^, ^37^, ^38^ but not yet applied to placebo‐controlled trials in psoriasis. Of note, simpler assessments based on single‐point colorimetry and average A* failed to show differences compared to placebo which highlights that internal controls might be warranted to limit intersubject variation. It is known that skin colour strongly impacts the visual representation of psoriatic lesions and therewith colour based assessments.^39^ Indeed, using multispectral imaging to derive redness from haemoglobin levels instead of CIELAB values resulted in an evident treatment effect compared to placebo, which might be attributed to a decreased influence of skin tone.^40^, ^41^ As results in this study are based on a population with predominantly Fitzpatrick skin type of II and III, translation to patients with skin types IV and higher may remain limited and should first be investigated. The high superficial haemoglobin levels are well‐supported by the observed increases in subcutaneous perfusion determined by LSCI at baseline and its subsequent normalization during guselkumab treatment. OCT measurements reiterated that acanthosis and rete ridges are observable through increased epidermal thickness and decreases during effective treatment,^42^, ^43^ now also in the target plaque of mild patients. However, epidermal thickness did not significantly decrease compared to placebo, which may be partly attributed to a small measurement surface and heterogeneity in the plaques themselves,^44^ or to substantial distortion of the OCT signal by rete ridges making the dermal–epidermal junction harder to locate.^45^ Together, these measures differentiate healthy and nonlesional skin from lesional skin and reflect treatment over time, thereby supporting their use in early clinical drug development.

Unfortunately, not all measures yielded favourable results as dPASI correlated poorly with the physician‐reported PASI despite previously having been validated in an observational study.^26^ However, validation of the dPASI was performed based on PASI‐scoring of images *posthoc*. Direct comparisons between the live physician‐performed PASI and dPASI yielded differences exceeding 40%. Similar variation in this study might have obscured treatment effects in a population with mild disease. Disregarding the often‐cited shortcomings of the physician‐performed PASI in favour of computerized substitutes, it highlights that this method remains insufficiently powered to detect effects to a level that is expected in a clinical trial setting. However, the advance of deep learning might offer new opportunities for the conception of a fully automized PASI but remains challenging.^46^

Although this study demonstrated the feasibility of using a mild psoriasis population in clinical trials with IL‐23 inhibition, the translatability to moderate‐to‐severe patients in future trials could be limited by differences in disease phenotype that could differentially affect outcomes. Although the genetic background has been shown to differ between mild and moderate‐to‐severe psoriasis,^47^ the transcriptome still shows a high degree of similarity.^13^, ^48^ Although a more apparent immune signature around IL‐17 was observed in mild disease, this did not seem to affect treatment responses.^14^

The ability to demonstrate treatment responses in a small cohort with low disease severity is promising for its application in early drug development. However, guselkumab is 1 of a variety of biologics that have collectively pushed the acceptable response rate in clinical care from PASI75 to PASI90.^49^, ^50^ Although target lesion monitoring would be ideal for following responses to topical therapies, these are often associated with weaker responses and might therefore not show such an evident effect as guselkumab therapy.^10^ The sensitivity to detect less evident changes using this toolbox remains to be explored. However, novel drug candidates for psoriasis will have to demonstrate equal or higher responses to achieve market approval and reimbursement by medical insurance.

## 5 CONCLUSION

Altogether, this study establishes mild psoriasis patients as a suitable and representative population for early phase clinical research. A high degree of confidence was obtained by bolstering traditional endpoints with novel objective outcomes. The possibility to conduct data‐rich trials in this mild population will facilitate easier patient recruitment and provide earlier signs of efficacy of candidate drugs in proof‐of‐concept studies.

## AUTHOR CONTRIBUTIONS

R.R., M.D. and J.R. designed the research study. J.R. and M.B. performed the research under supervision of M.D., R.R., M.D., J.B. and N.K. T.N., J.R., L.B., S.L., R.B. and Y.Y. analysed the data. J.R. wrote the first manuscript draft. M.B., M.D., R.R. and J.B. reviewed the manuscript. All authors have read and approved the manuscript.

## CONFLICT OF INTEREST STATEMENT

Dr van Doorn has received consulting fees or honorarium from Novartis, AbbVie, Pfizer, LEO pharma, Sanofi, Lilly, Janssen and Celgene and grant and payment for lectures including service on speakers bureaus from Novartis, Sanofi and Janssen outside the submitted work. The other authors have no conflicts of interest to declare.

## PATIENT CONSENT STATEMENT

Written informed consent, including the use of photographs, was obtained from all participants to participate in the study prior to any study‐related procedures.

## Supporting information


**FIGURE S1** Completed PRISM flowchart showing the number of patients screened, included and analysed.
**TABLE S1** Baseline demographics of the study population. Significant differences in psoriasis severity between the groups is presented for the psoriasis area and severity index (PASI), digital PASI (dPASI) and lesion severity score, but not for the Physicians Global Assessment (PGA). Contrasts are made compared to the mild guselkumab group. ns, *P >* .05; * *P <* .05; *** *P <* .0001.
**TABLE S2** Overview of adverse effects recorded during the study.
**TABLE S3** Statistical output of all longitudinal scores and assessments mentioned in the main text for change to baseline (all groups) and compared to placebo (mild‐GUS *vs.* mild‐PLA), separately. For change from baseline: the least square mean (LSM) change from baseline, followed by the 95% confidence interval and *P*‐value are shown. For comparisons between mild‐GUS *vs.* mild‐PLA, the LSM of mild‐GUS is contrasted with that of mild‐PLA, 95% confidence interval and the *P*‐value.
**FIGURE S2** All available datapoints of the physician‐performed psoriasis area and severity index (PASI) plotted against their digital PASI (dPASI) counterpart. Note that both the full PASI scores and the PASI scores without the head and neck area have been plotted, which might introduce bias as the scalp can be obscured by hair and therefore impact the dPASI assessments. A band has been plotted indicating the same score with physician PASI and dPASI with a datapoint at the middle‐dotted line. A ±2‐point margin is indicated in grey. Note that a repeated measures correlation is indicated in Table S4.
**FIGURE S3** results of superficial roughness analysis by multispectral imaging (A) and optical coherence tomography (B). Graphs show mean and standard deviation.
**FIGURE S4** Baseline differences and longitudinal time course during the trial of the degree of redness based on the CIELAB A* value determined by colorimetry (A), the average CIELAB A* value by multispectral imaging (B) and the delta CIELAB A*, being the difference between the lowest and highest recorded value within the region of interest, by multispectral imaging (C). Colorimetry did not indicate a difference compared to baseline and not compared to. Average A* was significantly lower compared to baseline in both guselkumab treated groups but not compared to placebo. Delta A* showed a significant decrease compared to baseline and also compared to placebo.
**FIGURE S5** Overview of the superficial texture as recorded with multispectral imaging of additional patients.
**FIGURE S6** Overview of erythema as recorded with multispectral imaging of additional patients.
**FIGURE S7** Overview of cutaneous perfusions as recorded with Laser Speckle Contrast Imaging of additional patients.
**FIGURE S8** Overview of a frame from an optical biopsy as recorded with optical coherence tomography of additional patients. A dotted line indicates the dermal–epidermal junction. ND indicates that the basal–epidermal junction could not be reliably determined in that frame of the scan.
**TABLE S4** Correlations in the guselkumab treated group, both mild and moderate‐to‐severe, between all modalities. The clinical scores comprised of the psoriasis area and severity index (PASI), PASI‐high discrimination (PASI‐HD) and the lesion severity score (LSS) are emphasized. Numerical data represent the Repeated Measure Correlation (r_rm_).
**TABLE S5** Correlations in the guselkumab treated group, both mild and moderate‐to‐severe, between subscores of the lesion severity score (LSS) and the objective modalities. Plausible connections between specific endpoints and lesion severity subscores (e.g. LSS—erythema and maximal erythema) are emphasized. Numerical data represent the repeated measure correlation (r_rm_).

## Data Availability

The data that support the findings of this study are available from the corresponding author upon reasonable request.
