# Supplementary material for: Mild psoriasis as a suitable model for proof‐of‐mechanism in a phase 1B setting: Results from a double‐blind placebo‐controlled trial with guselkumab
Source: Br J Clin Pharmacol. 2025 Jul 28;91(12):3378–88. doi: 10.1002/bcp.70179 (PMC12648370; doi:10.1002/bcp.70179)
Supplement: Supplementary file 1 — FIGURE S1 Completed PRISM flowchart showing the number of patients screened, included and analysed. TABLE S1 Baseline demographics of the study population. Significant differences in psoriasis severity between the groups is presented for the psoriasis area and severity index (PASI), digital PASI (dPASI) and lesion severity score, but not for the Physicians Global Assessment (PGA). Contrasts are made compared to the mild guselkumab group. ns, P > .05; * P < .05; *** P < .0001. TABLE S2 Overview of adverse effects recorded during the study. TABLE S3 Statistical output of all longitudinal scores and assessments mentioned in the main text for change to baseline (all groups) and compared to placebo (mild‐GUS vs. mild‐PLA), separately. For change from baseline: the least square mean (LSM) change from baseline, followed by the 95% confidence interval and P‐value are shown. For comparisons between mild‐GUS vs. mild‐PLA, the LSM of mild‐GUS is contrasted with that of mild‐PLA, 95% confidence interval and the P‐value. FIGURE S2 All available datapoints of the physician‐performed psoriasis area and severity index (PASI) plotted against their digital PASI (dPASI) counterpart. Note that both the full PASI scores and the PASI scores without the head and neck area have been plotted, which might introduce bias as the scalp can be obscured by hair and therefore impact the dPASI assessments. A band has been plotted indicating the same score with physician PASI and dPASI with a datapoint at the middle‐dotted line. A ±2‐point margin is indicated in grey. Note that a repeated measures correlation is indicated in Table S4. FIGURE S3 results of superficial roughness analysis by multispectral imaging (A) and optical coherence tomography (B). Graphs show mean and standard deviation. FIGURE S4 Baseline differences and longitudinal time course during the trial of the degree of redness based on the CIELAB A* value determined by colorimetry (A), the average CIELAB A* value by multispectral imag [file BCP-91-3378-s001.docx]

Supplemental information pertaining to “Mild psoriasis as a suitable model for proof-of-mechanism in a phase 1B setting; results from a double-blind placebo-controlled trial with guselkumab”

By Rousel, Bergmans *et al.* (2025)

**Overview of the in- and exclusion criteria for this study**

Eligible healthy controls must meet all of the following inclusion criteria at screening:

1. Male or non-pregnant female subjects, 18 to 75 years of age (inclusive); during COVID-19 pandemic this is set to 18 to 69 year of age (inclusive)
2. Healthy as defined by the absence of any uncontrolled active or uncontrolled chronic disease following a medical and surgical history, documentation of general symptoms, and a symptom-directed physical examination including vital signs;
3. Willing to give written informed consent and willing and able to comply with the study protocol;

And eligible healthy controls must meet none of the following exclusion criteria at screening:

1. History or symptoms of any uncontrolled, significant disease including (but not limited to), neurological, psychiatric, endocrine, cardiovascular, respiratory, gastrointestinal, hepatic, or renal disorder that may interfere with the study objectives, in the opinion of the Investigator;
2. History of immunological abnormality (e.g., immune suppression, severe allergy or anaphylaxis) that may interfere with study objectives, in the opinion of the Investigator;
3. Known infection requiring antibiotic therapy within the last three months prior to the study;
4. Immunosuppressive or immunomodulatory treatment within 30 days prior to the study;
5. Body mass index (BMI) ≤ 18.0 or ≥ 40.0 kg/m^2^; during COVID-19 pandemic only ≤ 18.0 or > 33.0 kg/m^2^
6. Participation in an investigational drug study within 3 months prior to screening or more than 4 times a year;
7. Previous participation in an investigational drug study involving the dosing of an investigational compound targeting an immune pathway within one year prior to screening;
8. Loss or donation of blood over 500 mL within three months prior to screening;
9. The use of any medication or vitamin/mineral/herbal/dietary supplement within less than 5 half-lives prior to study participation, if the Investigator judges that it may interfere with the study objectives. The use of paracetamol (up to 4 g/day) is allowed;
10. History of alcohol consumption exceeding 5 standard drinks per day on average within 3 months of screening. Alcohol consumption will be prohibited from at least 12 hours preceding each study visit;
11. Any other condition that could interfere with the conduct of the study or the study objectives, in the opinion of the Investigator.
12. During COVID-19 pandemic: presence of high risk comorbidities: such as cardiovascular, respiratory or immune system disorders

Eligible psoriasis patients must meet all of the following inclusion criteria at screening:

1. Male or non-pregnant female subjects, 18 to 75 years of age (inclusive); during COVID-19 pandemic this is set to 18 to 69 year of age (inclusive)
2. Diagnosed with plaque psoriasis at least 6 months prior to study participation
3. Willing to discontinue any psoriasis therapy other than emollients.
4. Having mild (PASI ≥1 and ≤ 5) or moderate-to-severe (PASI ≥ 10) plaque psoriasis;
5. Currently not using psoriasis medication and ≥ 2 plaques suitable for repeated biopsies and target lesion assessments. At least one of these lesions must be located on the extremities, preferably on the elbow or knee, with a minimal target lesion score between 6 and 9. Or, when currently using psoriasis medication and insufficient lesional skin is present, willing to discontinue treatment awaiting rescreening (see also exclusion criteria 3 for psoriatic patients);
6. Willing to give written informed consent and willing and able to comply with the study protocol;

And none of the following exclusion criteria at screening:

1. Having primarily erythrodermic, pustular or guttate psoriasis;
2. Having medication-induced psoriasis;
3. Having previously failed on anti-IL23 therapy;
4. Having received treatments for psoriasis within the following intervals prior to the start of the study:
5. < 2 weeks for topical treatment, e.g. retinoids, corticosteroids, vitamin D analogs
6. < 4 weeks for phototherapy, e.g. PUVA, PDT
7. < 4 weeks for non-biologic systemic treatment, e.g. retinoids, methotrexate, cyclosporine, fumaric acid esters
8. < 4 weeks for etanercept
9. < 8 weeks for adalimumab
10. < 3 months for anti-IL17, anti-IL12(/23) and anti-IL23 treatments
11. History or symptoms of any significant uncontrolled disease including (but not limited to), neurological, psychiatric, endocrine, cardiovascular, respiratory, gastrointestinal, hepatic, or renal disorder that may interfere with the study objectives, in the opinion of the Investigator, excluding psoriasis and conditions that are related to psoriasis;
12. History of immunological abnormality (e.g., immune suppression, severe allergy or anaphylaxis) that may interfere with study objectives, in the opinion of the Investigator;
13. Known infection requiring antibiotic therapy within the last 3 months prior to the study, including latent tuberculosis;
14. Systemic immunosuppressive or immunomodulatory treatment within 30 days prior to the study;
15. Body mass index (BMI) ≤ 18.0 or ≥ 40.0 kg/m^2^; during COVID-19 pandemic only ≤ 18.0 or > 33.0 kg/m^2^
16. Participation in an investigational drug study within 3 months prior to screening or more than 4 times a year;
17. Loss or donation of blood over 500 mL within three months prior to screening;
18. The use of any medication or vitamin/mineral/herbal/dietary supplement within less than 5 half-lives prior to study participation, if the Investigator judges that it may interfere with the study objectives. The use of paracetamol (up to 4 g/day) and is allowed;
19. History of alcohol consumption exceeding 5 standard drinks per day on average within 3 months of screening. Alcohol consumption will be prohibited from at least 12 hours preceding each study visit;
20. Any other condition that could interfere with the conduct of the study or the study objectives, in the opinion of the Investigator.
21. During COVID-19 pandemic: presence of high risk comorbidities: such as cardiovascular, respiratory or immune system disorders other than psoriasis and psoriasis arthritis

**Extended methods**

Treatment allocation was performed by a study-independent statistician. Randomization was performed in blocks of four, separately for sex and severity. As placebo and guselkumab were supplied in visually different syringes, administration of guselkumab and placebo was performed by study-independent physicians that did not partake in other clinical activities surrounding the trial, administration was performed in a separate room and subjects were instructed to look away during administration. Therefore, the study team and patients remained blinded, and the double blinded nature of the trial was upheld. The study was ended in January 2023 as all mild patients and healthy controls had been recruited.

Supplemental figure s1: Completed PRISM flowchart showing the number of patients screened, included and analyzed.

Supplemental table s1: Baseline demographics of the study population. Significant differences in psoriasis severity between the groups is presented for the psoriasis area and severity index (PASI), digital PASI (dPASI) and lesion severity score, but not for the Physicians Global Assessment (PGA). Contrasts are made compared to the mild guselkumab group. ns; p>0.05, *; p<0.05,***; p<0.0001.

|  |  | **Controls** | **Mild (placebo)** | **Mild (Guselkumab)** | **Moderate-to-severe (Guselkumab)** |
| --- | --- | --- | --- | --- | --- |
| Total number of patients | | 10 | 5 | 15 | 5 |
| Age at first dose | ≤ 18 years | 0 (0%) | 0 (0%) | 0 (0%) | 0 (0%) |
|  | 18 - 65 years | 10 (100%) | 5 (100%) | 14 (93%) | 5 (100%) |
|  | ≥ 65 years | 0 (0%) | 0 (0%) | 1 (7%) | 0 (0%) |
| Sex | Female | 7 (70%) | 1 (20%) | 3 (20%) | 2 (40%) |
|  | Male | 3 (30%) | 4 (80%) | 12 (80%) | 3 (60%) |
| Race | White | 9 (90%) | 4 (80%) | 13 (87%) | 4 (80%) |
|  | Asian | 0 (0%) | 1 (20%) | 0 (0%) | 0 (0%) |
|  | Hispanic | 1 (10%) | 0 (0%) | 0 (0%) | 1 (20%) |
|  | More than one | 0 (0%) | 0 (0%) | 2 (13%) | 0 (0%) |
| Fitzpatrick | I | 0 (0%) | 0 (0%) | 1 (7%) | 0 (0%) |
|  | II | 5 (50%) | 2 (40%) | 5 (33%) | 3 (60%) |
|  | III | 4 (40%) | 2 (40%) | 9 (60%) | 1 (20%) |
|  | IV | 1 (10%) | 0 (0%) | 0 (0%) | 1 (20%) |
|  | V | 0 (0%) | 0 (0%) | 0 (0%) | 0 (0%) |
|  | VI | 0 (0%) | 1 (20%) | 0 (0%) | 0 (0%) |
| Baseline PASI (mean ± SD) | | n.a. | 4.5±2.3^ns^ | 5.6±2.0 | 12.5±0.9*** |
| Baseline dPASI (mean ± SD) | | n.a. | 4.0±1.9^ns^ | 4.6±1.5 | 6.8±1.7*--- |
| Baseline PASI-HD (mean ± SD) | | n.a. | 3.3±2.2^ns^ | 2.2±2.4 | 11.7±2.7*** |
| Baseline Lesion Severity Score  (mean ± SD) | | n.a. | 6.8±1.3^ns^ | 7.1±0.9 | 6.8±1.3^ns^ |
| Baseline PGA | 0 (Clear) | n.a. | 0 (0%) | 0 (0%) | 0 (0%) |
|  | 1 (Almost clear) | n.a. | 0 (0%) | 0 (0%) | 0 (0%) |
|  | 2 (Mild) | n.a. | 3 (60%) | 15 (100%) | 1 (20%) |
|  | 3 (Moderate) | n.a. | 2 (40%) | 0 (0%) | 4 (60%) |
|  | 4 (Severe) | n.a. | 0 (0%) | 0 (0%) | 1 (20%) |

Supplemental table s2: Overview of adverse effects recorded during the study.

|  |  | Mild (Placebo) | Mild (Guselkumab) | Moderate-to-severe (Guselkumab) |
| --- | --- | --- | --- | --- |
| Total Adverse Events |  | 16 (5/5) | 22 (11/15) | 5 (4/5) |
| Eye disorders | Glaucoma | 0 (0/5) | 1 (1/15) | 0 (0/5) |
| Gastrointestinal disorders | Gastrointestinal viral infection | 1 (1/15) | 1 (1/15) | 1 (1/5) |
|  | Diarrhea | 3 (2/5) | 1 (1/15) | 0 (0/5) |
|  | Abdominal pain | 0 (0/5) | 1 (1/15) | 0 (0/5) |
| General disorders and administration site conditions | Fatigue | 0 (0/5) | 2 (2/15) | 0 (0/5) |
|  | Fever | 0 (0/5) | 1 (1/15) | 0 (0/5) |
|  | Injection site erythema | 0 (0/5) | 1 (1/15) | 0 (0/5) |
| Infections and infestations | SARS-CoV-2 infection | 4 (4/5) | 3 (3/15) | 1 (1/5) |
|  | Influenza | 2 (2/5) | 0 (0/15) | 0 (0/5) |
|  | Upper respiratory tract infections | 1 (1/5) | 2 (2/15) | 1 (1/5) |
|  | Vaginal yeast infection | 1 (1/5) | 0 (0/15) | 0 (0/5) |
|  | Impetigo | 0 (0/5) | 1 (1/15) | 0 (0/5) |
| Injury poisoning and procedural complications | Scapula fracture | 0 (0/15) | 0 (0/15) | 1 (1/5) |
|  | Rib contusion | 0 (0/15) | 0 (0/15) | 1 (1/5) |
|  | Traumatic hematoma | 1 (1/5) | 0 (0/15) | 0 (0/5) |
|  | Post vaccination syndrome | 0 (0/5) | 1 (1/15) | 0 (0/5) |
|  | Scarring | 0 (0/5) | 1 (1/15) | 0 (0/5) |
| Musculoskeletal and connective tissue disorders | Muscular back pain | 0 (0/5) | 0 (0/5) | 1 (1/5) |
| Nervous systems disorders | Headache | 1 (1/5) | 4 (4/15) | 0 (0/5) |
| Skin and subcutaneous tissue disorders | Alopecia | 1 (1/5) | 1 (1/15) | 0 (0/5) |
|  | Allergic dermatitis | 0 (0/5) | 1 (1/15) | 0 (0/5) |
|  | Pruritus | 1 (1/5) | 1 (1/15) | 0 (0/5) |
| Surgical and medical procedures | Dental operation | 1 (1/5) | 0 (0/15) | 0 (0/5) |

Supplemental table s3: Statistical output of all longitudinal scores and assessments mentioned in the main text for change to baseline (all groups) and compared to placebo (mild-GUS versus mild-PLA), separately. For change from baseline: the least square mean (LSM) change from baseline, followed by the 95% confidence interval and p-value are shown. For comparisons between Mild-Gus versus Mild-PLA, the LSM of Mild-GUS is contrasted with that of Mild-PLA, 95% confidence interval and the p-value.

|  | ***Change from baseline*** | | | ***End of study*** |
| --- | --- | --- | --- | --- |
|  | **Mild patients (GUS)** | **Moderate-to-severe patients (GUS)** | **Mild patients (PLA)** | **Mild patients (GUS)  vs  mild patients (PLA)** |
| Clinical scoring | | | | |
| **PASI** | -5.52 (-6.43 to -4.60) p<0.0001 | -9.03 (-11.12 to -6.93) p<0.0001 | -0.84 (-2.45 to 0.77) p=0.30 | 2.56 vs 5.75 (-4.61 to -1.75) p=0.0001 |
| **PASI-HD** | -3.71 (-4.53 to -2.90) p<0.0001 | -7.96 (-9.96 to -5.97) p<0.0001 | 0.46 (-0.96 to 1.87) p=0.52 | 1.84 vs 4.21 (-3.64 to -1.11) p=0.0008 |
| **Lesion severity score** | -5.62 (-6.47 to -4.77) p<0.0001 | -6.67 (-8.14 to -5.20) p<0.0001 | 0.84 (-0.72 to 2.41) p=0.29 | 2.7 vs 6.8 (-5.4 to -2.7) p<0.0001 |
| **dPASI** | -0.92 (-1.53 to -0.30) p=0.0042 | -2.59 (-3.70 to -1.48) p<0.0001 | -0.11 (-1.16 to 0.93) p=0.83 | 3.61 to 4.27 (-1.63 to 0.30) p=0.17 |
| Colorimeter | | | | |
| **Average A* (AU)** | -0.29 (-2.32 to 1.74) p=0.78 | -2.26 (-5.83 to 1.30) p=0.21 | -0.33 (-3.83 to 3.17) p=0.85 | 18.62 vs 17.67 (-1.81 to 3.71) p=0.48 |
| Multispectral imaging | | | | |
| **Roughness (mm)** | 3.54 (-6.27 to -0.81) p=0.012 | -3.89 (-8.61 to 0.83) p=0.11 | -2.88 (-7.62 to 1.86) p=0.23 | 12.85 vs 15.35 (-6.45 to 1.45) p=0.20 |
| **Maximal Height (mm)** | -0.083 (-0.12 to -0.043) p<0.0001 | -0.089 (-0.16 to -0.021) p=0.011 | -0.039 (-0.11 to 0.031) p=0.27 | 0.19 vs 0.27 (-0.15 to -0.18) p=0.014 |
| **Average A* (AU)** | -1.00 (-2.00 to 0.00) p=0.051 | -2.49 (-4.25 to -0.73) p=0.0063 | -0.75 (-2.49 to 1.00) p=0.40 | 11.15 to 11.41 (-1.82 to 1.28) p=0.72 |
| **Delta A* (AU)** | -1.21 (-1.60 to 0.82) p<0.0001 | -1.20 (-1.87 to -0.53) p=0.0006 | -0.36 (-1.04 to 0.31) p=0.28 | 2.37 vs 3.14 (-1.28 to -0.25) p=0.0055 |
| **Maximal Erythema (AU)** | -16.85 (-22.36 to -11.34) p<0.0001 | -16.72 (-26.32 to -7.12) p=0.0009 | 2.34 (-7.28 to 11.96) p=0.6294 | 47.17 vs 61.23 (-22.10 to -6.02) p=0.0015 |

|  | ***Change from baseline*** | | | ***End of study*** |
| --- | --- | --- | --- | --- |
|  | **Mild patients (GUS)** | **Moderate-to-severe patients (GUS)** | **Mild patients (PLA)** | **Mild patients (GUS)  vs  mild patients (PLA)** |
| Optical coherence Tomography | | | | |
| **Rougness (µm)** | -0.0045 (-0.0085 to -0.00045) p=0.030 | -0.0064 (-0.012 to -0.00078) p=0.026 | -0.0027 (-0.016 to 0.010) p=0.68 | 0.018 to 0.22 (-0.011 to 0.0023) p=0.20 |
| **Epidermal thickness (mm)** | -0.10 (-0.13 to -0.074) p<0.0001 | -0.13 (-0.18 to -0.084) p<0.0001 | -0.038 (-0.087 to 0.011) p=0.13 | 0.21 vs 0.25 (-0.084 to 0.002) p=0.061 |
| Laser Speckle Contrast Imaging | | | | |
| **Perfusion (AU)** | -46.57 (-58.97 to -34.18) p<0.0001 | -62.29 (-83.72 to -40.86) p<0.0001 | 0.34 (-21.25 to 21.93) p=0.98 | 74.41 vs 107.12 (-50.87 to 14.55) p=0.0012 |
| Thermography | | | | |
| **Skin temperature (°C)** | -0.30 (-1.06 to 0.46) p=0.44 | -0.18 (-1.41 to 1.05) p=0.77 | -0.46 (-1.78 to 0.86) p=0.49 | 32.05 vs 32.26 (-1.32 to 0.89) p=0.69 |


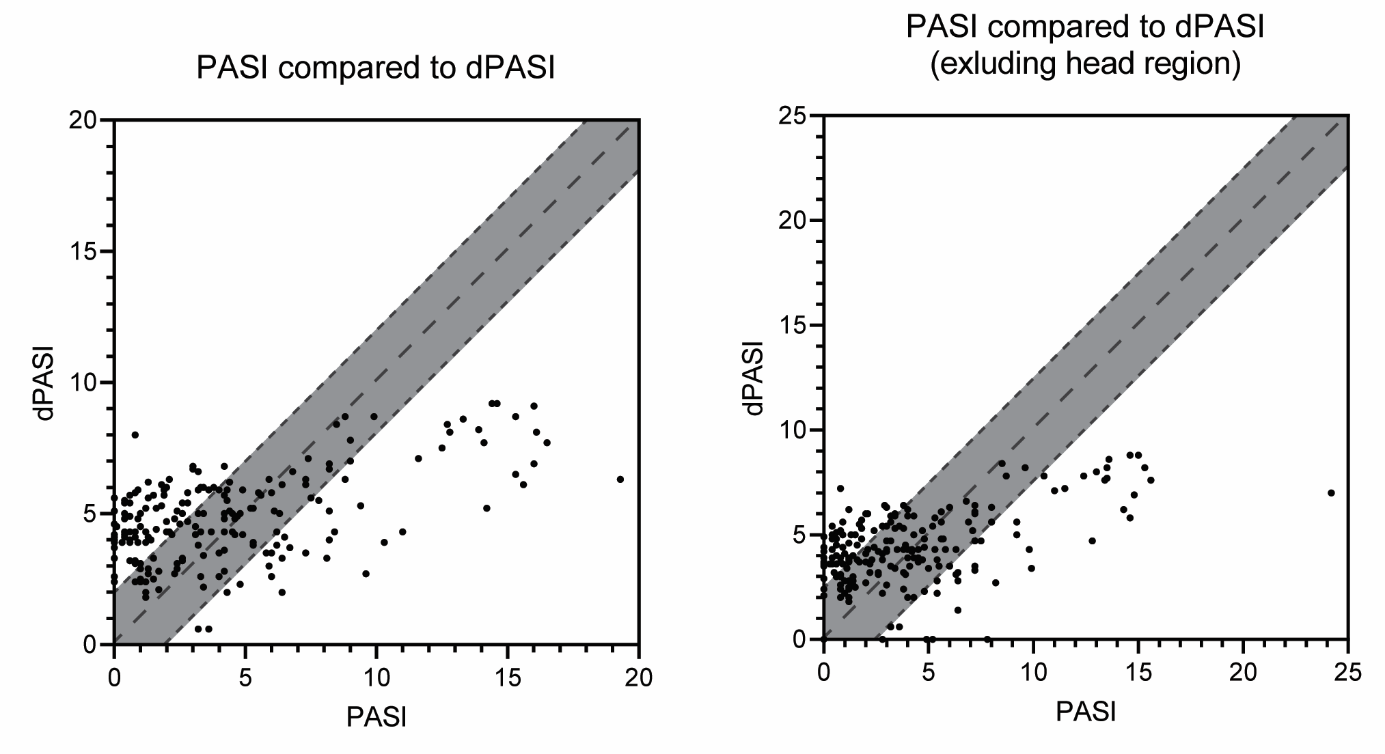
Supplemental figure s2: All available datapoints of the physician-performed psoriasis area and severity index (PASI) plotted against their digital PASI (dPASI) counterpart. Note that both the full PASI scores and the PASI scores without the head and neck area have been plotted, which might introduce bias as the scalp can be obscured by hair and therefore impact the dPASI assessments. A band has been plotted indicating the same score with physician PASI and dPASI with a datapoint at the middle-dotted line. A ±2-point margin is indicated in grey. Note that a repeated measures correlation is indicated in supplemental table s4.
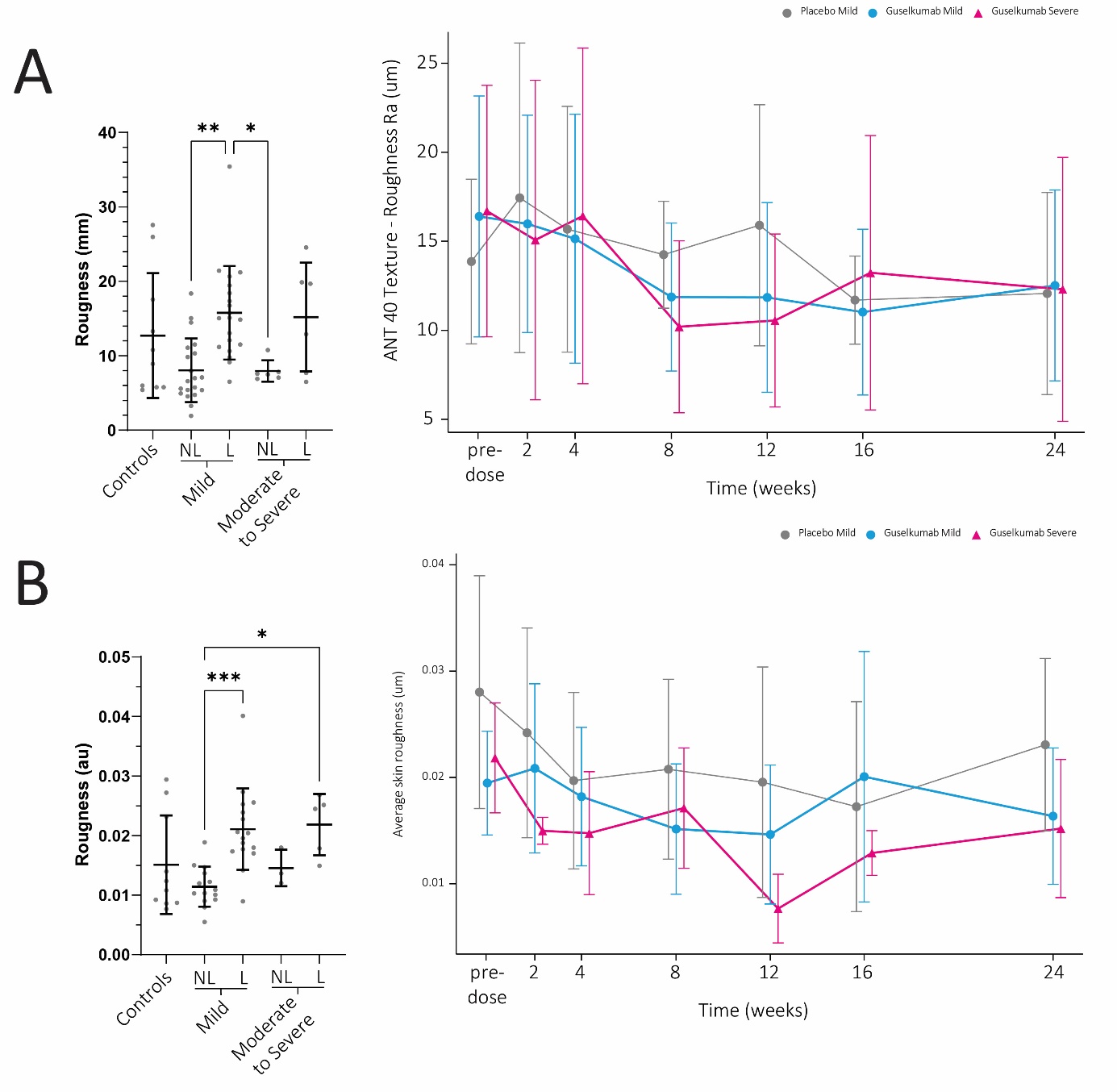
Supplemental figure s3: results of superficial roughness analysis by multispectral imaging (a) and optical coherence tomography (b). Graphs show mean and standard deviation.


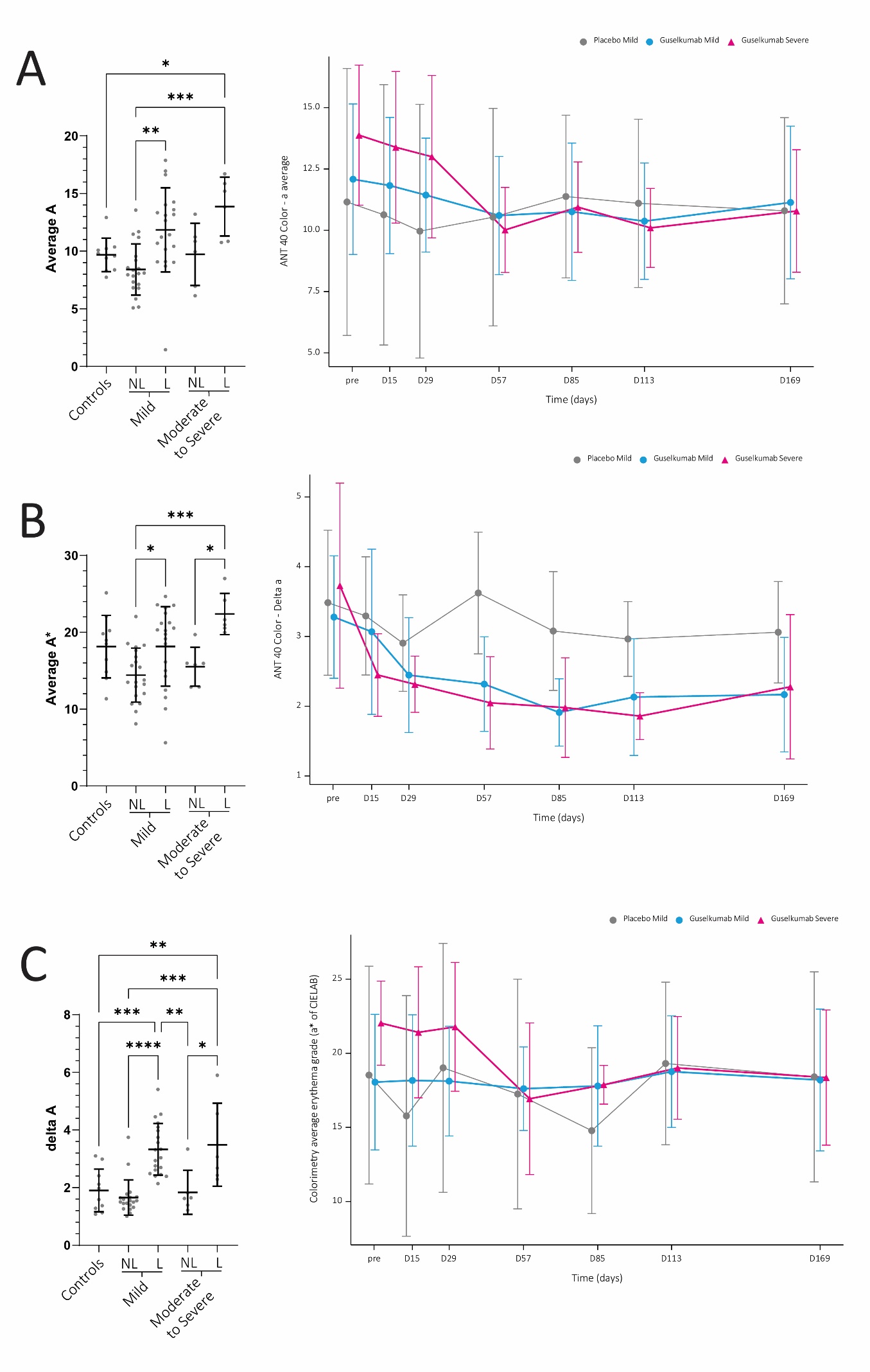


Supplemental figure s4: Baseline differences and longitudinal time course during the trial of the degree of redness based on the CIELAB A* value determined by colorimetry (A), the average CIELAB A* value by multispectral imaging (B) and the delta CIELAB A*, being the difference between the lowest and highest recorded value within the region of interest, by multispectral imaging (C). Colorimetry did not indicate a difference compared to baseline and not compared to. Average A* was significantly lower compared to baseline in both guselkumab treated groups but not compared to placebo. Delta A* showed a significant decrease compared to baseline and also compared to placebo.


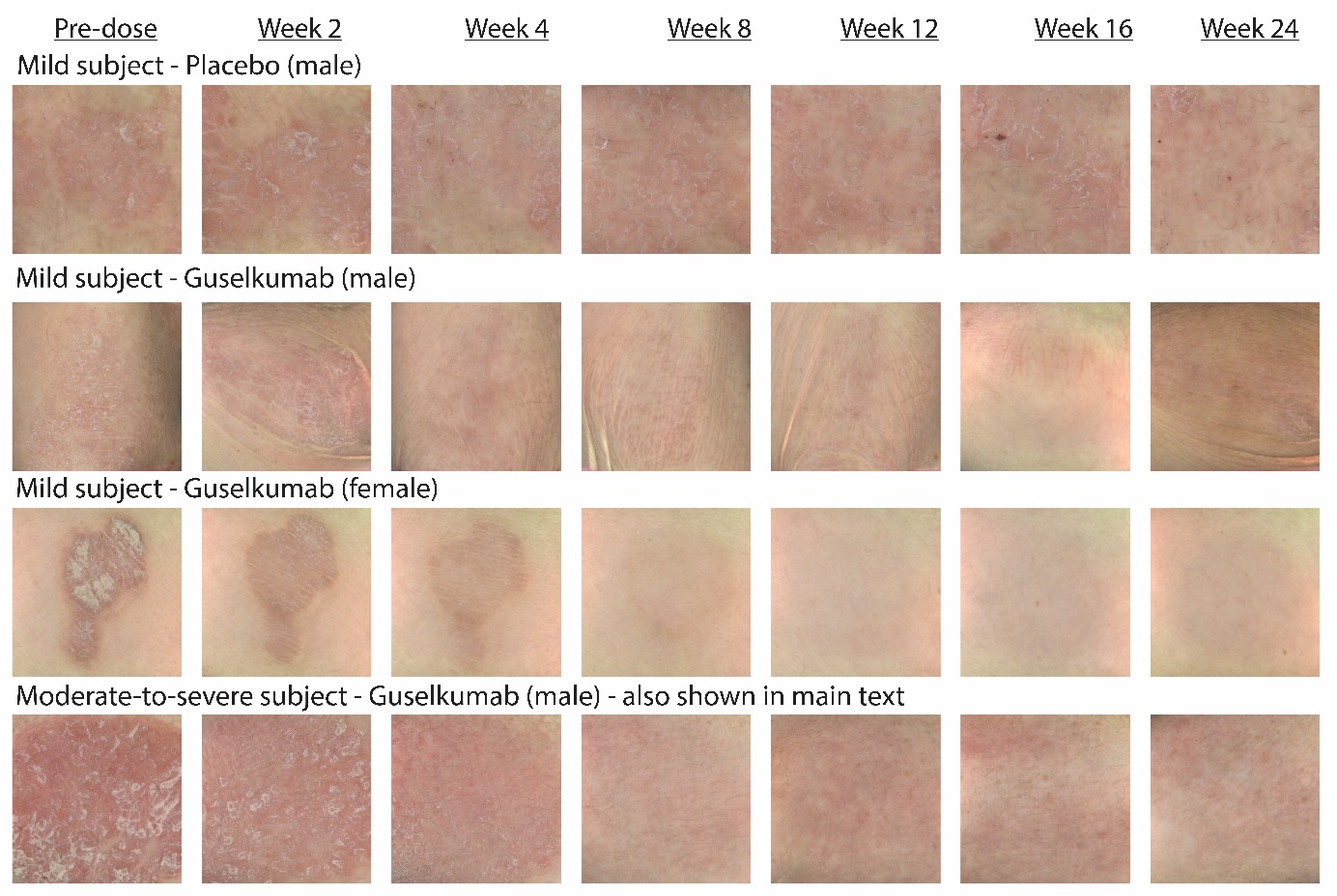
Supplemental figure s5: Overview of the superficial texture as recorded with multispectral imaging of additional patients.
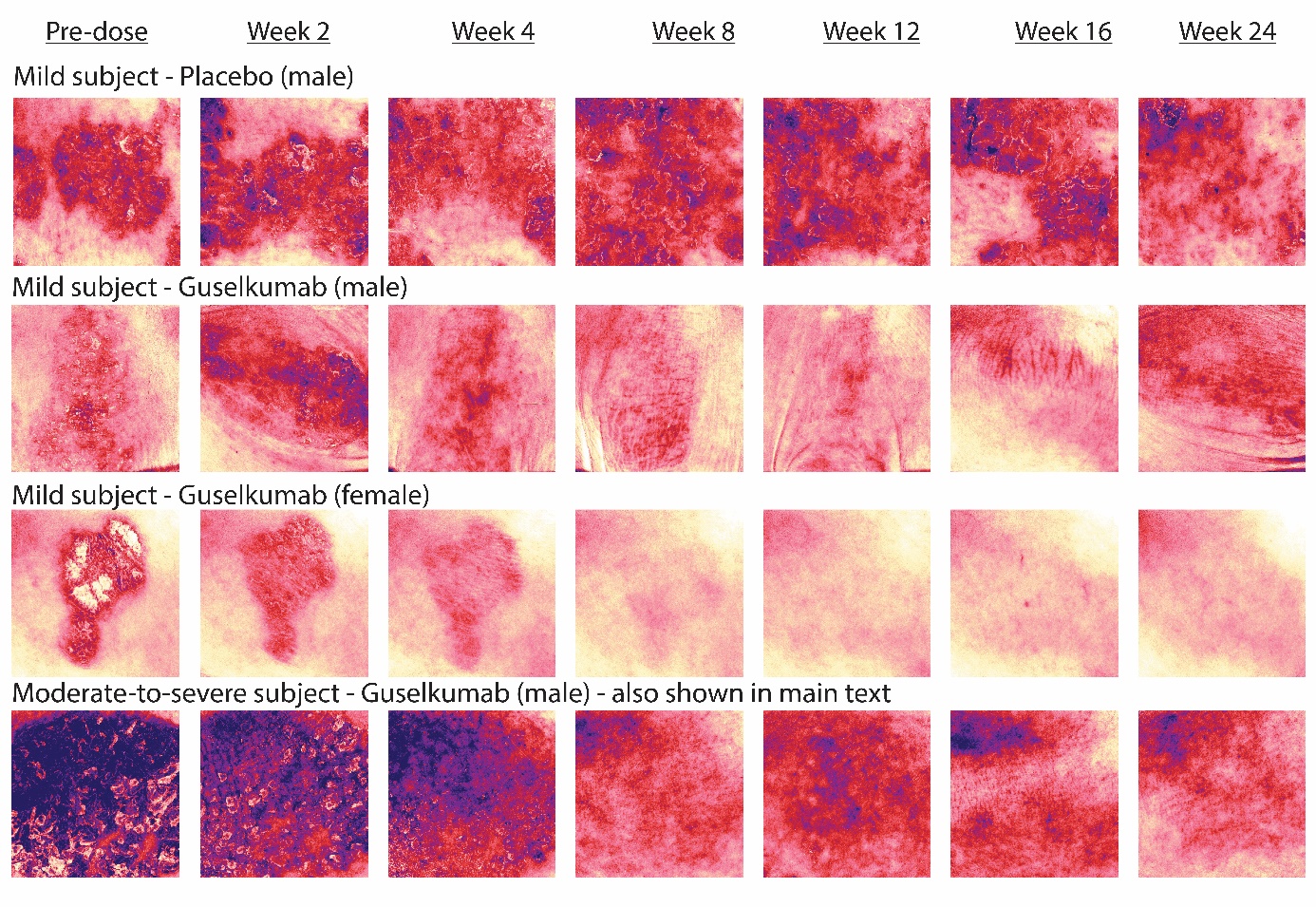
Supplemental figure s6: Overview of erythema as recorded with multispectral imaging of additional patients.


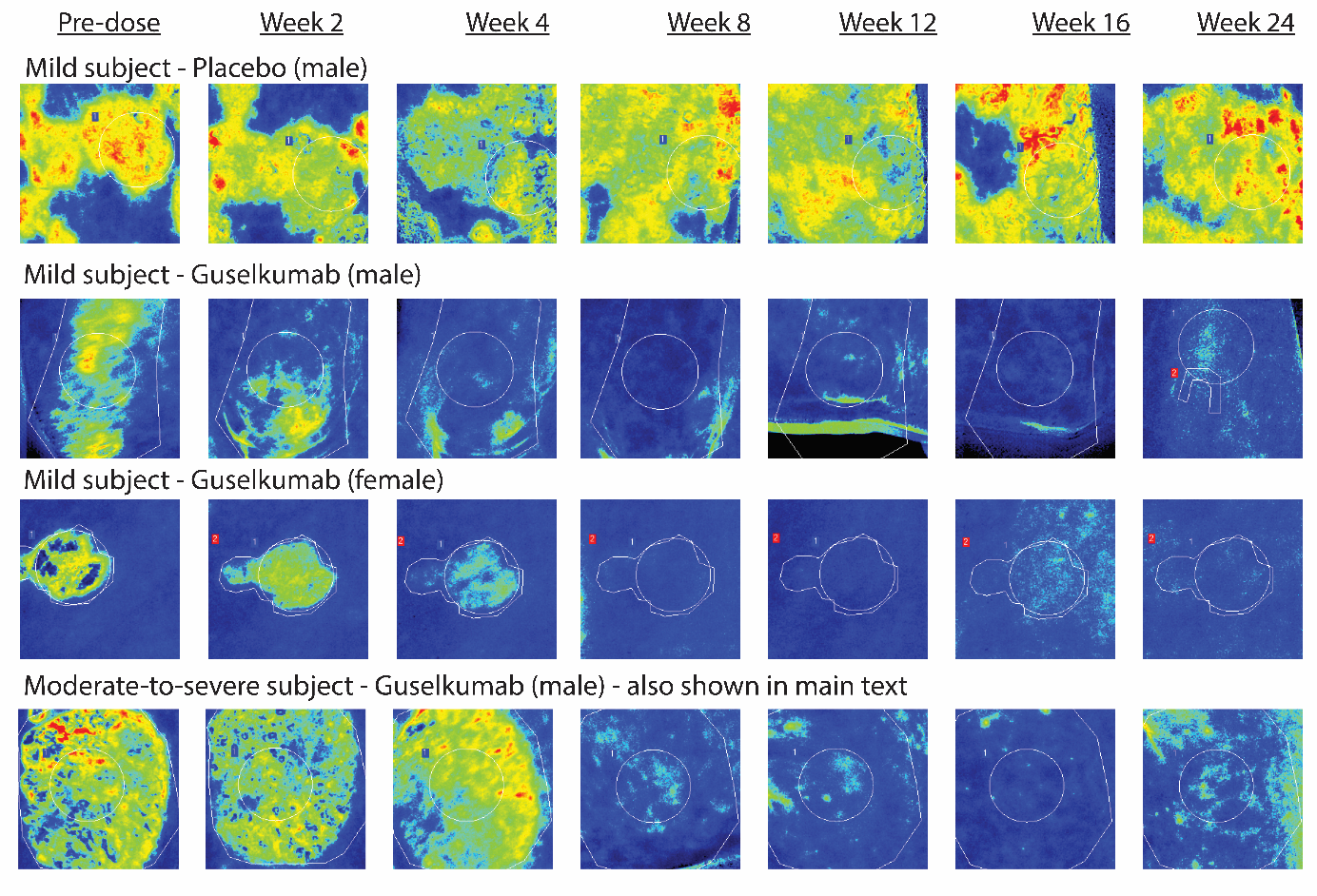
Supplemental figure s7: Overview of cutaneous perfusions as recorded with Laser Speckle Contrast Imaging of additional patients.


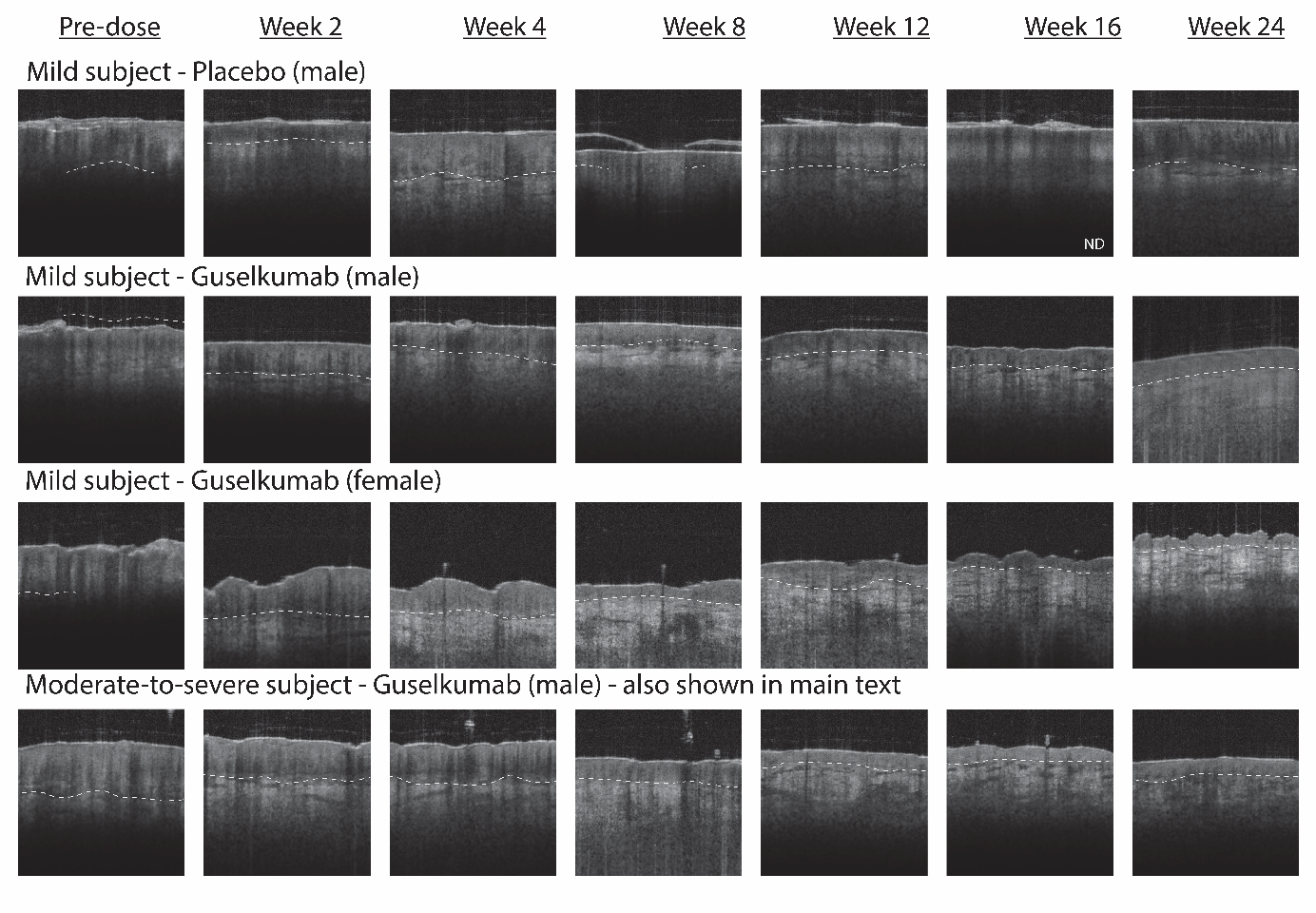


Supplemental figure s8: Overview of a frame from an optical biopsy as recorded with optical coherence tomography of additional patients. A dotted line indicates the dermal-epidermal junction. ‘ND’ indicates the basal-epidermal junction could not be reliably determined in that frame of the scan.

Supplemental table s4: Correlations in the guselkumab treated group, both mild and moderate-to-severe, between all modalities. The clinical scores comprised of the Psoriasis Area and Severity Index (PASI), PASI-High Discrimination (PASI-HD) and the Lesion Severity Score (LSS) are emphasized. Numerical data represent the Repeated Measure Correlation (r_rm_).

|  | PASI | PASI-HD | Lesion Severity Score (LSS) | Digital PASI | Basal Flow (mm/sec) | Epidermal Thickness (mm) | Maximal Erythema | Maximal Height (mm) |
| --- | --- | --- | --- | --- | --- | --- | --- | --- |
| PASI | 1.00 | 0.95 | 0.85 | 0.45 | 0.63 | 0.71 | 0.58 | 0.45 |
| PASI-HD | 0.95 | 1.00 | 0.74 | 0.51 | 0.57 | 0.68 | 0.52 | 0.37 |
| Lesion Severity Score (LSS) | 0.85 | 0.74 | 1.00 | 0.33 | 0.67 | 0.68 | 0.69 | 0.56 |
| Digital PASI | 0.45 | 0.51 | 0.33 | 1.00 | 0.31 | 0.40 | 0.23 | 0.15 |
| Basal Flow (mm/sec) | 0.63 | 0.57 | 0.67 | 0.31 | 1.00 | 0.44 | 0.58 | 0.45 |
| Epidermal Thickness (mm) | 0.71 | 0.68 | 0.68 | 0.40 | 0.44 | 1.00 | 0.47 | 0.33 |
| Maximal Erythema | 0.58 | 0.52 | 0.69 | 0.23 | 0.58 | 0.47 | 1.00 | 0.49 |
| Maximal Height (mm) | 0.45 | 0.37 | 0.56 | 0.15 | 0.45 | 0.33 | 0.49 | 1.00 |

Supplemental table s5: Correlations in the guselkumab treated group, both mild and moderate-to-severe, between subscores of the Lesion Severity Score (LSS) and the objective modalities. Plausible connections between specific endpoints and lesion severity subscores (e.g. LSS – Erythema and maximal erythema) are emphasized. Numerical data represent the Repeated Measure Correlation (r_rm_).

|  | Lesion Severity Score (LSS) | LSS - Erythema | LSS - Scaling | LSS - Induration |
| --- | --- | --- | --- | --- |
| Lesion Severity Score (LSS) | 1.00 | 0.91 | 0.94 | 0.93 |
| Basal Flow (mm/sec) | 0.67 | 0.54 | 0.64 | 0.68 |
| Maximal Erythema | 0.69 | 0.60 | 0.63 | 0.69 |
| Maximal Height (mm) | 0.56 | 0.41 | 0.60 | 0.53 |
| Epidermal Thickness (mm) | 0.68 | 0.59 | 0.64 | 0.66 |

**Supplemental references**

1. Jaspers, M. E. H. & Moortgat, P. Objective Assessment Tools: Physical Parameters in Scar Assessment. *Textbook on Scar Management* 149–158 (2020) doi:10.1007/978-3-030-44766-3_17.
